# Identification of COUP-TFII Orphan Nuclear Receptor as a Retinoic Acid–Activated Receptor

**DOI:** 10.1371/journal.pbio.0060227

**Published:** 2008-09-16

**Authors:** Schoen W Kruse, Kelly Suino-Powell, X. Edward Zhou, Jennifer E Kretschman, Ross Reynolds, Clemens Vonrhein, Yong Xu, Liliang Wang, Sophia Y Tsai, Ming-Jer Tsai, H. Eric Xu

**Affiliations:** 1 Laboratory of Structural Sciences, Van Andel Research Institute, Grand Rapids, Michigan, United States of America; 2 Department of Physics, Grand Valley State University, Allendale, Michigan, United States of America; 3 Global Phasing Ltd., Sheraton House, Castle Park, Cambridge, United Kingdom; 4 Department of Molecular and Cellular Biology and Program in Developmental Biology, Baylor College of Medicine, Houston, Texas, United States of America; Baylor College, United States of America

## Abstract

The chicken ovalbumin upstream promoter-transcription factors (COUP-TFI and II) make up the most conserved subfamily of nuclear receptors that play key roles in angiogenesis, neuronal development, organogenesis, cell fate determination, and metabolic homeostasis. Although the biological functions of COUP-TFs have been studied extensively, little is known of their structural features or aspects of ligand regulation. Here we report the ligand-free 1.48 Å crystal structure of the human COUP-TFII ligand-binding domain. The structure reveals an autorepressed conformation of the receptor, where helix α10 is bent into the ligand-binding pocket and the activation function-2 helix is folded into the cofactor binding site, thus preventing the recruitment of coactivators. In contrast, in multiple cell lines, COUP-TFII exhibits constitutive transcriptional activity, which can be further potentiated by nuclear receptor coactivators. Mutations designed to disrupt cofactor binding, dimerization, and ligand binding, substantially reduce the COUP-TFII transcriptional activity. Importantly, retinoid acids are able to promote COUP-TFII to recruit coactivators and activate a COUP-TF reporter construct. Although the concentration needed is higher than the physiological levels of retinoic acids, these findings demonstrate that COUP-TFII is a ligand-regulated nuclear receptor, in which ligands activate the receptor by releasing it from the autorepressed conformation.

## Author Summary

Unlike other classes of receptors, nuclear receptors can bind directly to DNA and act as transcription factors, playing key roles in embryonic development and cellular metabolism. Most nuclear receptors are activated by signal-triggering molecules (ligands) and can regulate their activity by recruiting coactivator proteins. However, the ligands are unknown for a subset of “orphan” nuclear receptors, including the chicken ovalbumin promoter-transcription factors (COUP-TFI and II, and EAR2). COUP-TFs are the most conserved nuclear receptors, with roles in angiogenesis, neuronal development, organogenesis, and metabolic homeostasis. Here we demonstrate that COUP-TFII is a ligand-regulated nuclear receptor that can be activated by unphysiological micromolar concentrations of retinoic acids. We determined the structure of the ligand-free ligand-binding domain of the human COUP-TFII, revealing the autorepressed conformation of the receptor, where helix α10 is bent into the ligand-binding pocket and the activation function-2 helix is folded into the cofactor binding site, thus preventing the recruitment of coactivators. These results suggest a mechanism where ligands activate COUP-TFII by releasing the receptor from the autorepressed conformation. The identification of COUP-TFII as a low-affinity retinoic acid receptor suggests ways of searching for the endogenous ligands that may ultimately link retinoic acid and COUP-TF signaling pathways.

## Abstract 3

Structural and functional studies reveal that the orphan nuclear receptor COUP-TFII is a low-affinity receptor for retinoic acids. paving the way to finding the endogenous ligands that may ultimately link retinoic acid and COUP-TF signaling pathways.

## Introduction

Nuclear receptors (NRs) are ligand-inducible transcription factors that transmit physiological signals of a wide variety of ligands, such as classical steroid hormones, retinoic acid, thyroid hormone, and vitamin D [1,2]. The NR family also includes a large number of orphan receptors for which specific ligands have yet to be identified [3]. Among the most extensively studied orphan receptors are the chicken ovalbumin upstream promoter-transcription factors (COUP-TFs), which belong to the NR2F subfamily. This family includes three human members—COUP-TFI (EAR3), COUP-TFII (ARP-1), and the more distant EAR2—as well as the Drosophila melanogaster protein Seven-up (Svp), xCOUP-TFIII from Xenopus laevis, and the zebrafish homolog SVP46 [4,5]. COUP-TFs are the most evolutionarily conserved NRs among all species, and within the NR2F subfamily, the homology in both the DNA-binding domain (DBD) and ligand-binding domain (LBD) is extremely high. For example, the LBDs of COUP-TFI or II are essentially identical in different species (99.6% among vertebrates and >90% with the D. melanogaster protein Svp), suggesting that these domains are critical for the biological function of COUP-TFs even though a ligand has yet to be identified [4].

In mammals, the COUP-TF orphan NRs regulate many key biological processes, including angiogenesis, neuronal development, organogenesis, cell fate determination, metabolic homeostasis, and circadian rhythm [6–12]. *COUP-TFII–*null mutants exhibit defects in angiogenesis and heart development and die before embryonic day 10.5 [7]. COUP-TFII also regulates vein identity by repressing Notch signaling [13]. In addition, *COUP-TFII* heterozygous females show significantly reduced fertility, irregular estrus cycles, delayed puberty, and retarded postnatal growth [14]. Conditional deletion of COUP-TFII in the uterus results in decidualization and embryo attachment defects, leading to infertility [15], whereas partial ablation of COUP-TFII causes severely impaired placental formation and contributes to miscarriage [16]. Tissue-specific knockouts of *COUP-TFII* in the mesenchyme cause an alteration in the anterior-posterior and radial patterning of the stomach and causes Bochdalek-type congenital diaphragmatic hernia [17,18]. Altogether, the role of COUP-TFII during angiogenesis and heart development, female reproduction, and mesenchymal-epithelial signaling has been well established, even though it is unclear whether COUP-TFII is regulated by ligands.

The LBD of NRs plays a crucial role in their functions, including ligand recognition, receptor dimerization or oligomerization, and ligand-dependent activation. Crystallographic studies have revealed that NR activity is primarily determined by the conformational states of the activation function-2 (AF2) helix located at the C terminus of the LBD [19]. In the agonist-bound receptor, the AF2 helix is stabilized in an active conformation to form a charge-clamp for interaction with coactivator LXXLL motifs [20–22]. These structures show that the LXXLL coactivator motif adopts a two-turn α helix with the three leucine side chains fitting into a hydrophobic pocket between two charge-clamp residues that cap both helical ends. In contrast to the coactivator-bound structures, the longer LXXXIXXXL/I corepressor motif adopts a three-turn α helix and forces the AF2 helix to shift conformations to make room for the larger motif, thereby disrupting the coactivator binding groove [23]. Alternatively, antagonists can also bind to LBDs and promote an “autoinhibited” conformation. The structure of the estrogen receptor α (ERα) in complex with the antagonist 4-hydroxytamoxifen (OHT) shows the AF2 helix binding in the coactivator binding site, rendering the LBD incapable of binding to coactivators [21,24]. While a large number of ligand-bound NR structures have been determined, few structures of NR LBDs exist in the absence of ligands [20,25]. The structures of apo-RXRα have been solved as both a dimer and tetramer, and both structures show the AF2 helix extending away from the core domain of the LBD [26,27]. In the apo-RXRα tetramer, the AF2 helix of each monomer spans into the coactivator binding site in the adjacent monomer of the symmetric dimer, therefore forming an auto-repressed complex where the AF2 helix physically blocks LBD interactions with coactivators or corepressors [27]. These studies highlight the importance of structural biology in revealing novel insights into NR ligand binding and cofactor interactions. Elucidation of a COUP-TF LBD structure is crucial for understanding how this important subfamily of receptors is regulated.

Here we report the 1.48 Å crystal structure of the LBD of human COUP-TFII. This structure represents a novel structure of an auto-inhibited NR, a conformation where the intramolecular interaction between the AF2 helix and the cofactor binding site physically blocks the interaction with either coactivators or corepressors. We also use cell-based activation assays to identify coactivators that enhance COUP-TFII activation and residues that play a role in ligand binding, cofactor recruitment, and dimerization. Furthermore, we provide evidences that retinoid acids can promote the ability of COUP-TFII to interact with coactivator motifs, and to activate a COUP-TF reporter construct. These observations establish that COUP-TFII is a ligand-regulated NR and reveal a structural mechanism that ligand-dependent activation of COUP-TFII is in part mediated through the release of the receptor from the auto-repression state.

## Results

### Structure Determination and Crystal Structure of the COUP-TFII LBD

The human COUP-TFII LBD was purified to homogeneity in a ligand-free state (see Methods). Although it has been shown that the inclusion of LXXLL motifs is crucial for the crystallization of a number of NR LBD complexes [20,28–30], we crystallized the COUP-TFII LBD in the absence of cofactor peptides. Molecular replacement solutions were obtained using the structure of the 9-cis retinoic acid–bound RXRα LBD [29] because of its 45% sequence homology to COUP-TFII, but these solutions failed to produce an interpretable electron density map for the lower third of the protein, including the bottom portion α10 and the AF2 helix. As a result, independent phase information was determined by multiple isomorphous replacement with data from derivative crystals containing iodine, yielding a clear structure for majority of the missing regions of COUP-TFII. There is one LBD molecule per asymmetric unit, but COUP-TFII forms a symmetric dimer through crystal packing. The data collection and refinement statistics are shown in Table 1.

**Table 1:**
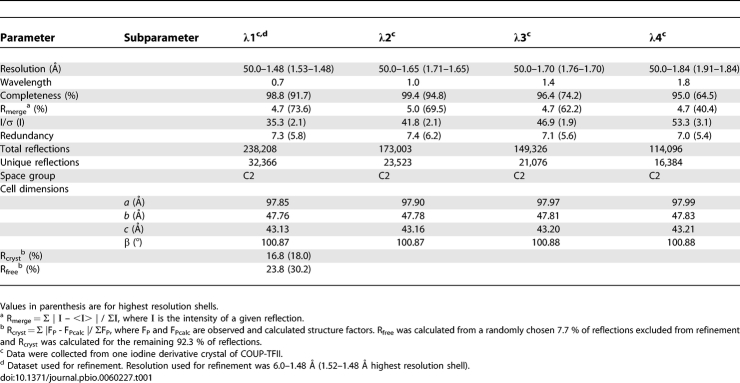
Data Collection and Refinement Statistics

**Figure 1:**
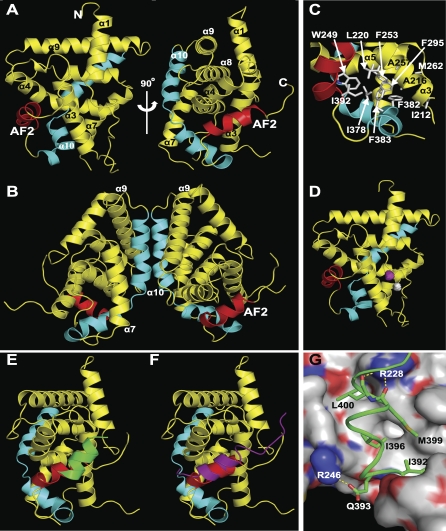
Crystal Structure of the Ligand-Free COUP-TFII LBD (A) Front and side views of the COUP-TFII LBD monomer with its AF2 helix colored in red. (B) Organization of the COUP-TFII LBD dimer, showing that its dimer interface is formed predominantly by helix α10 (cyan). (C) The packing of the ligand-binding pocket within the bottom half of the COUP-TFII LBD. (D) Space-filling diagram shows two small cavities in COUP-TFII colored with magenta (18 Å^3^) and white (12 Å^3^). (E and F) Overlay of the COUP-TFII LBD structure with the SRC-1 LXXLL motif (green in E) from the RXR structure or with the SMRT corepressor motif (magenta in F) from the antagonist bound PPARα structure. (G) Hydrogen bonds (yellow dashed lines) and hydrophobic interactions of the COUP-TFII AF2 helix (green) within the cofactor binding site.

Figure 1A shows two views of the overall structure of the COUP-TFII LBD monomer. The structure contains 10 α helices that are folded into a typical three-layered helical sandwich seen in other NRs. In the structure, two COUP-TFII monomers packed against each other to form a dimer, with its overall dimer configuration resembling the RXR homodimers or heterodimers (Figure 1B). The COUP-TFII LBD dimer buries 975 Å^2^ of surface area and is formed primarily by residues from helices α10 (cyan), α9, α8, and α7, as well as the loop between α8 and α9. The dimer interface is made up of residues involved in hydrophobic interactions and hydrogen bonding (Table 2), with the majority of the hydrophobic interactions observed between residues found on the N-terminal half of helix α10 of each monomer, which forms a parallel coiled-coil structure in the crystal. Most residues in the interface between helices α7, α9, and the loop between α8 and α9 are charged and are primarily involved hydrogen bonding.

**Table 2:**
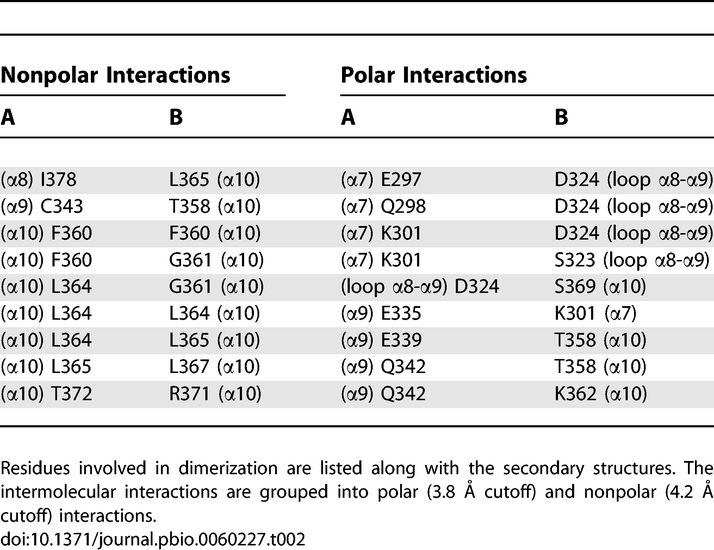
Interactions between COUP-TFII A and B

In the absence of ligand, helix α10 bends at V373 and causes the C-terminal portion of α10 to collapse into the lower half of the receptor, the region where ligands have been found to bind in other NR LBDs [30]. While the top half of α10 is involved in the dimer interface, the lower half folds into the ligand-binding pocket, preventing the binding of ligands and possibly contributing to the stability of the ligand-free state of the protein (Figure 1A). In contrast to the structure of RXRα bound to 9-cis retinoid acid (9cRA), where the binding pocket is occupied by the ligand and helix α10 is fully extended [27], the structure of COUP-TFII shows that the ligand binding site is occupied by hydrophobic and aromatic residues from α3 (I212, A216, L220), α5 (W249, F253, A257), the loop following α5 (M262), α7 (F295), α10 (I378, F382, F383), and from the AF2 helix (I392) (Figure 1C). Due to the bulky size of these aromatic side chains and the dense pack of the binding pocket in COUP-TFII, there is no room for any ligand to bind in this conformation. In fact, when calculating available cavity size in this structure, two small cavities were identified with volumes of 18 Å^3^ and 12 Å^3^ in size (magenta and white, respectively, Figure 1D) [31]. In comparison, the volume of a single methyl group is approximately 25 Å^3^, and based on this structure, the cavities in COUP-TFII would be too small to accommodate a ligand of this size.

The kink in helix α10 and the subsequent collapse of the binding pocket of COUP-TFII allows the AF2 helix, which follows α10, to bind in the cofactor binding site of the LBD. The sequence IETLIRDML from COUP-TFII AF2 helix (residues 392–400, where underlined residues are identical or similar to leucine or isoleucine) is highly related to the LXXLL coactivator motif or the LXXXIXXXL corepressor motif, and its binding mode resembles that of the coactivator SRC-1 peptide motif bound to RXRα from the RXRα/PPARγ heterodimer [29] or the corepressor silencing mediator of retinoid and thyroid receptor (SMRT) peptide from the PPARα-GW6471 structure [23] (Figure 1E and 1F). The AF2 helix is stabilized in the cofactor binding site by both hydrogen bonding and hydrophobic interactions. The N-terminal end of AF2 is stabilized by a hydrogen bond between Q393 (AF2) and R246 (α4), and the C-terminal end of the AF2 is stabilized by hydrogen bonding between the conserved charge clamp residue R228 (α3) and two backbone carbonyl groups from residues M399 and L400 (AF2) (Figure 1G). These hydrogen bonds lock the AF2 in place at the ends of the helix, while hydrophobic interactions help stabilize AF2 in the cofactor binding groove. I392, I396, M399, and L400 extend directly into the core of COUP-TFII and make Van der Waals contacts with residues from α3, α4, α5, and α10 (Figure 1G). In this orientation of the AF2 helix, neither coactivators nor corepressors are able to bind to COUP-TFII, and therefore this structure represents an autorepressed form of this orphan NR.

### COUP-TFII Acts as a Transcriptional Activator in Multiple Cell Lines

**Figure 2:**
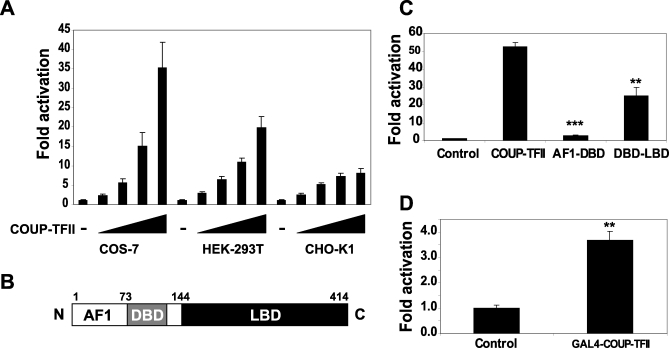
COUP-TFII Acts as an Activator of Transcription in Multiple Cell Lines (A) Activation of the *NGFI-A* promoter reporter construct with increasing concentrations of COUP-TFII (0ng, 50ng, 100ng, 150ng, and 200 ng of the expression vector, respectively, for each cell line). (B) Domain structure of COUP-TFII. (C) Effects of the COUP-TFII deletion mutants on activation of the *NGFI-A* promoter-driven reporter. The AF1-DBD construct activates ∼2-fold above empty vector control. (D) Activation by the GAL4-DBD-COUP-TFII-LBD. The fold activation is the relative fly luciferase activity of the *NGFI-A* promoter induced by COUP-TFII versus the control vector without COUP-TFII. All data are normalized to the activity of *Renilla* luciferase that was used as transfection control. For statistical analysis, the fold induction was compared with full-length COUP-TFII or GAL4-DBD in (C) and (D), respectively.

COUP-TFII can serve as an transcriptional activator of the *NGFI-A* promoter in HeLa and rat urogenital mesenchymal cells [32] and enhance hepatocyte nuclear factor 4 (HNF4)-induced cholesterol 7α-hydroxylase expression via a direct repeat one site [33]. To correlate the observed structure with COUP-TFII function, we established a cell-based assay using a full-length COUP-TFII expression construct and a luciferase reporter driven by the *NGFI-A* promoter in COS-7, HEK-293T, and CHO-K1 cells. Results showed a dose-dependent increase in gene expression in all three different cell types (Figure 2A), demonstrating the ability of COUP-TFII to activate the *NGFI-A* promoter in multiple cell lines.

The full-length COUP-TFII sequence consists of 414 amino acids and can be subdivided based on primary structure into the AF1 domain, the DBD, and the LBD (Figure 2B). To determine the specific contribution of each domain in COUP-TFII activation, we tested the transcriptional activity of a series of deletion mutants in cell-based assays. Removal of the AF1 domain (residues 1–73) resulted in a decrease of COUP-TFII activity of approximately 50% compared to wild-type levels, although the presence of the DBD and LBD alone are enough to activate gene expression by 25-fold over empty vector control (Figure 2C). Removal of the LBD, however, reduced more than 90% activity of COUP-TFII in our cell-based assay system and implies that the LBD is required to bind to ligands or coactivator proteins, or both, to activate transcription (Figure 2C). To test the activity of the LBD only, the COUP-TFII LBD (residues 144–414) was fused to the GAL4 DNA binding domain and cotransfected with a GAL4 reporter vector in COS-7 cells. The GAL4-COUP-TFII chimera construct activated luciferase transcription greater than 3.5-fold over GAL4 DBD alone (Figure 2D), indicating that the COUP-TFII LBD alone is adequate to activate gene transcription.

### COUP-TFII Activation Requires the Formation of a Functional Dimer and the AF2 Helix

**Figure 3:**
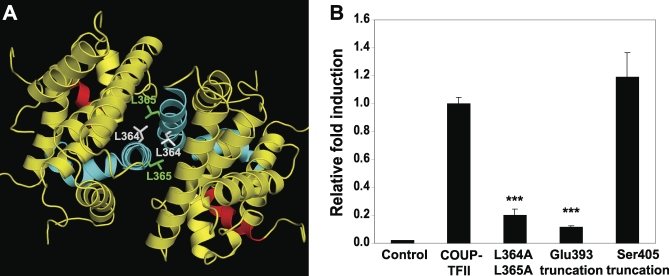
COUP-TFII Activation Is Dependent on the Formation of a Functional Dimer and the Presence of AF2 (A) Top view of the COUP-TFII dimer showing the close packing of L364 (gray) and L365 (green) from helices α10 (cyan) in the dimer interface. (B) Effects of the L364A/L365A double mutant and the AF2 deletion mutant on COUP-TFII activation of the *NGFI-A* promoter. For easy comparison, the relative fold of activation by the wild-type receptor is set to 1. The statistical analysis for the fold induction of the mutants was compared with wild type COUP-TFII.

The COUP-TFII LBD forms a symmetric dimer along helix α10 of each monomer. To determine the functional role of the COUP-TFII dimer, we mutated two leucines (L364 and L365) from the N-terminal portions of helix α10 to alanines. These two leucines are key interface residues that form critical hydrophobic interactions with I318, G361, L364, L365, and L367 of the opposite monomer (Figure 3A and Table 2). The L364A/L365A double mutant showed only 20% activity in comparison to wild-type COUP-TFII, indicating that an intact dimer interface is required for COUP-TFII to function properly (Figure 3B). These data support the initial studies of COUP-TF that showed the functional DNA-binding form of COUP-TF is a dimer [34,35].

To test the role of the AF2 helix in COUP-TFII activity, we made two truncation mutants at the C terminus. Truncation at position S405, which removes the C-terminal nine residues but keeps the AF2 helix intact, has little effect on the COUP-TFII transcriptional activity. In contrast, truncation at position E393, which removes the entire AF2 helix and all residues thereafter, causes a dramatic and significant loss of function of the receptor (Figure 3B), indicating that an intact AF2 helix is required for the COUP-TFII transcriptional function.

### Coactivators Bind to COUP-TFII via a Charge Clamp and Enhance Activation

**Figure 4:**
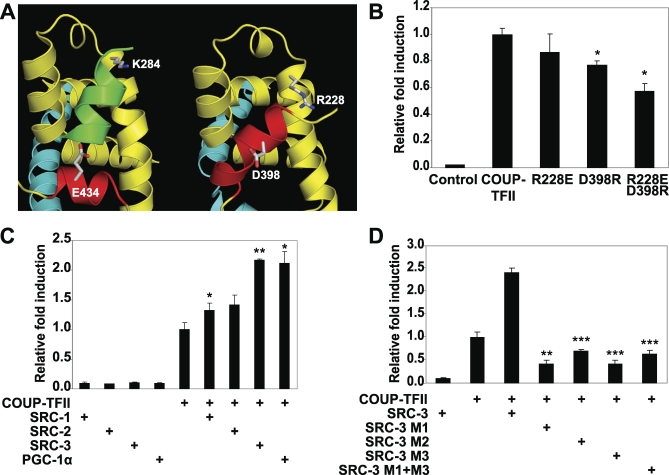
Coactivators Bind to COUP-TFII via a Charge Clamp and Enhance Activation (A) Comparison of the RXRα charge clamp (K284 and E434 in the left) with that of COUP-TFII (right). The SRC-1 LXXLL motif is shown in green. (B) Effects of the charge clamp mutations on COUP-TFII activation. The relative fold of activation by the wild-type receptor is set to 1 in (B), (C), and (D). The statistical analysis for the fold induction of the mutants was compared with wild-type COUP-TFII. (C) Effects of coactivators on COUP-TFII activation. The statistical analysis for the fold induction by coactivators was compared with the wild-type COUP-TFII in the absence of additional coactivators. (D) Effects of mutations in the three conserved SRC-3 LXXLL coactivator binding motifs (M1-M3) to LXXAA on the SRC-3-mediated enhancement of COUP-TFII induction. For statistical analysis, the fold induction was compared with COUP-TFII in (B) and (C) and COUP-TFII and SRC-3 cotransfection in (D). The statistical analysis for the fold induction by mutated coactivators was compared with that of wild type SRC-3.

Coactivator recruitment for transcriptional activation by NRs is mediated through a conserved charge clamp pocket, in part formed by a positively charged residue from the end of helix α3 and a negatively charged residue from the center of AF2 helix [19]. The charge clamp residues in COUP-TFII are R228 from helix α3 and D398 from the AF2 helix; both point away from the protein molecule (Figure 4A). To test the significance of the charge clamp in COUP-TFII activation, we mutated these two residues and tested them in cell-based activation assays. While single mutations of D398R and R228E have weak effects on COUP-TFII activation, complete removal of the charge clamp by the combined mutation reduces activation to 40% in comparison to the wild-type receptor (Figure 4B). These data show that an intact charge clamp is required to interact with endogenous coactivators for enhancing gene expression at wild-type levels.

Having shown a wild-type charge clamp capable of interacting with coactivators is important in COUP-TFII activity, we attempted to identify cofactor proteins that may enhance this activation. Previous studies have shown that the coactivators SRC-1 and GRIP1/SRC-2 can potentiate the activity of COUP-TFI both in vivo and upstream of the *NGFI-A* promoter in HeLa cells, and that PGC-1α and COUP-TFI interact with each other on the phosphoenolpyruvate carboxykinase (PEPCK) gene promoter [32,36,37]. Transfection of the coactivators SRC-1, SRC-2, SRC-3, and PGC-1α alone into COS-7 cells does not cause expression of luciferase downstream of the *NGFI-A* promoter (Figure 4C). However, when full-length COUP-TFII was cotransfected with these coactivators, almost all coactivators caused a significant increase in the relative induction of genes compared with COUP-TFII transactivation alone (Figure 4C). Specifically, both SRC3 and PGC-1α caused the most significant increase in the induction of luciferase (greater than 2-fold), suggesting that these coactivators play a role in COUP-TFII–mediated gene transcription, as they are found to be co-expressed with COUP-TFII in multiple tissues [15,38].

The coactivator SRC-3 (also called AIB1, ACTR, RAC-3, and TRAM-1) contains three highly conserved NR box LXXLL motifs (M1–M3) to mediate ligand-dependent interactions with NRs [39–42]. After identifying that SRC-3 enhances COUP-TFII-mediated transcription by more than 2-fold, we made a series of mutations at the conserved LXXLL motifs to LXXAA to disrupt this interaction and tested these mutations in cell-based assays. Mutations at each of the three motifs individually or as a combined M1–M3 mutation reduced COUP-TFII induction below that of wild-type, full-length receptor alone (Figure 4D). These data reveal that COUP-TFII can interact with each of the LXXLL motifs of SRC-3 and that disruption of any one of these motifs significantly reduces the SRC-3–mediated COUP-TFII transcription.

### An Intact Pocket Is Important for COUP-TFII Activation

**Figure 5:**
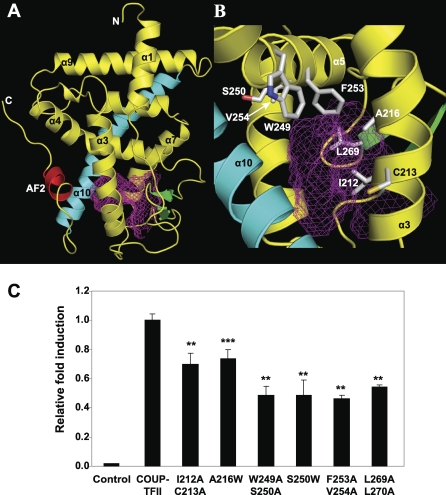
An Intact Pocket Is Important for COUP-TFII Activation (A) An active model of COUP-TFII. This model is based on the agonist-bound RXR structure with its helix α10 (cyan) extended and AF2 (red) in the active conformation. One cavity (magenta) with size of 659 Å^3^ is found in this conformation. (B) The potential ligand binding pocket (magenta mesh) in the active model of the COUP-TFII LBD and its surrounding residues. (C) Effects of pocket residue mutations on COUP-TFII activation. The relative fold of activation by the wild-type receptor is set to 1. The statistical analysis for the fold induction of the mutants was compared with wild-type COUP-TFII.

**Figure 6:**
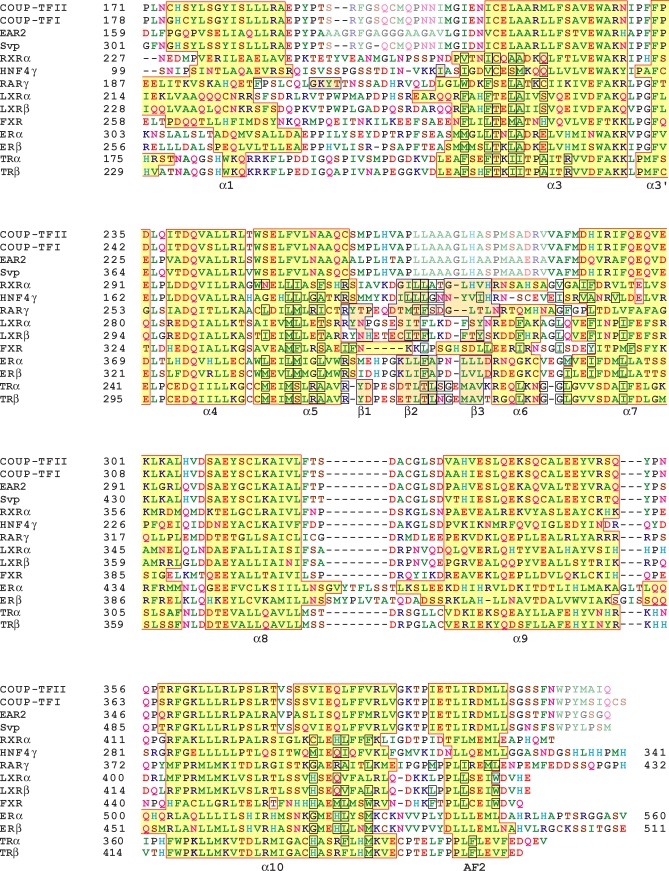
Conserved Positions of the Ligand Pocket Residues in NRs Structure-based sequence alignment of various NR LBDs shows that ligand pocket residues (boxed by black squares) are conserved in their relative positions within the context of their secondary structural elements (labeled underneath) of NRs. All sequences are from human proteins except Seven-up, a COUP-TF-like orphan receptor from D. melanogaster. The Protein Databank (PDB; http://www.rcsb.org/pdb/home/home.do) codes for the ligand/receptor complexes is: 1fmr for 9cRA-bound RXRα [28], 1lv4 for HNF4γ [61], 2lbd for RARγ [62], 1uhl for LXRα [63], 1pld for LXRβ [64], 1ot7 for FXR [65], 1l2i for ERα [21], 1qkm for ERβ [66], 2h79 for TRα[67], and 1q4x for TRβ [68,69].

The ligand-binding pocket of the apo-COUP-TFII structure is packed tightly with hydrophobic residues that leave little space for the binding of small molecules due to the kink of helix α10 (Figure 1). However, a sizeable cavity (∼600–700 Å^3^) for ligand binding was created when we built an active model of the COUP-TFII where helix α10 is straightened (Figure 5A). A straight helix α10 has been observed for all agonist-bound NR LBD structures, including the active structure of RXRα, where 9-cis-retinoid acid straightens helix α10 from its kink conformation in the apo-structure [27,29]. In addition, analysis of the existing crystal structures of several NR/ligand complexes and structural based sequence alignment reveals that ligand-contacting residues in NR LBDs are highly conserved in their relative positions within the primary sequence (boxed residues Figure 6). Inspection of the ligand-binding pocket of the active COUP-TFII model reveals that the residues at the above conserved positions indeed surround the COUP-TFII ligand-binding pocket with most of their side chains pointing toward the interior of the pocket (Figure 5B). Based on this information, we made a series of mutations in several residues that line the binding pocket in the active model of the COUP-TFII LBD, and we tested these mutations in cell-based assays.

Six sets of mutations were made to affect COUP-TFII ligand binding. Four sets of mutations were designed to increase the size of the ligand-binding pocket by mutating the corresponding residues to alanine (the double mutants I212A/C213A, W249A/S250A, F253A/V254A, and L269A/L270A), whereas two mutations were designed to reduce the size of the ligand-binding pocket with mutations to tryptophan residues (A216W and S250W). All mutations showed a significant decrease in activity in comparison to the wild-type receptor (Figure 5C). Two mutants showed a 30% decrease in activity (I212A/C213A and A216W), and four mutants reduced activity of COUP-TFII by 50% (W249A/S250A, S250W, F253A/V254A, and L269A/L270A). The degree of reduction in these mutants is comparable to the mutations in the ligand-binding pocket of SF-1, which was found to bind to phospholipids [30,43]. These results thus suggest that COUP-TFII may also be a ligand regulated receptor, which requires its intact binding pocket for the optimal receptor activity.

### Activation of COUP-TFII by Retinoid Acids

**Figure 7:**
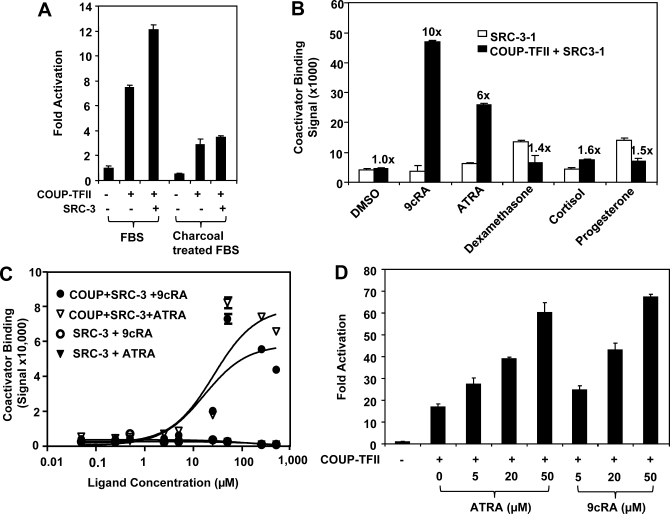
COUP-TFII Is Activated by Retinoid Acids (A) Effects of charcoal-treated FBS on COUP-TFII activation in the presence or absence of SRC-3 coactivator. The basal activity of the *NGFI-A* reporter construct in the presence of FBS and absence of COUP-TFII and SRC-3 is set as 1. (B) Addition of 50 μM of 9cRA or ATRA to promote COUP-TFII binding to SRC-3–1 coactivator motif where the addition of steroids (50 μM) has little effect. (C) Concentration-response curves of 9cRA and ATRA, which show the binding affinity (EC50) of 9cRA and ATRA is 17 μM and 26 μM, respectively. (D) Effects of ATRA and 9cRA on COUP-TFII activation of the NGFI-A promoter in COS-7 cells.

The transcriptional activity of COUP-TFII in multiple cell lines versus the autorepressed conformation observed in the apo-COUP-TFII structure suggests a putative ligand either present in the serum or produced in cell lines used. To test whether there is a COUP-TFII ligand in the serum, we repeated the activation experiment with dextran-charcoal–treated serum in the hope that such treatment would strip any hydrophobic ligands including steroids and retinoids, thus reducing COUP-TFII activation. Indeed, using charcoal-treated serum greatly reduced COUP-TFII activation potential by 60%–70% regardless the presence of the SRC-3 coactivator (Figure 7A), suggesting the presence of a hydrophobic ligand(s) in the serum, which is required for COUP-TFII activation.

The modeled active COUP-TFII conformation displays a ligand-binding cavity with a size of 600–700 Å^3^, which can easily adopt a steroid or retinoid ligand (Figure 5A). To determine the identity of possible COUP-TFII ligands, we screened a panel of steroids and retinoids for their ability to promote COUP-TFII to recruit the SRC-3–1 LXXLL coactivator motif. Both 9-cis-retinoid acid (9cRA) and all-trans-retinoid acid (ATRA) can enhance COUP-TFII to interact with the SRC-3–1 coactivator motif, while several steroids show little effect (Figure 7B). Full dose curves reveal the potency (EC50) of retinoid acids around 10–30 μM (Figure 7C). In parallel, both 9cRA and ATRA activate COUP-TFII on the luciferase reporter driven by the *NGFI-A* promoter with a similar potency of 20 μM (Figure 7D). Although the concentrations of RAs required for activation of COUP-TFII are 10–100 times higher than the physiological levels, these results nevertheless establish COUP-TFII is a ligand-activated receptor and demonstrate that both 9cRA and ATRA can serve as low-affinity ligands of COUP-TFII.

## Discussion

We have solved the structure of the COUP-TFII LBD, which reveals a novel autorepressed conformation of NRs crystallized in the absence of ligands. In contrast, cell-based assays indicate that COUP-TFII has a “constitutive” activity on the *NGFI-A* promoter, which can be further potentiated by recruiting coactivators like SRC-3 that require the LXXLL motifs. These two seemingly contrasting observations are reconciled by the fact that the active COUP-TFII model contains a ligand-binding pocket of 600–700 Å^3^, which can easily adopt a steroid or retinoid ligand. In addition, both 9cRA and ATRA bind and promote COUP-TFII activation. These results demonstrate that COUP-TFII is a ligand-regulated NR, whose full activity requires the intact structure of the COUP-TFII coactivator binding site, AF2 helix, dimer interface, and the residues that make up the COUP-TFII ligand-binding pocket. Moreover, the ability of 9cRA and ATRA to activate COUP-TFII in high concentrations indicates that RAs are unlikely to be the physiological ligands. Identification of the true endogenous ligands will require further research, which could help to reveal the ligand-dependent signaling pathways of the COUP-TF subfamily of orphan NRs.

### Structural Basis for the COUP-TFII Autorepressed Conformation

The classic mechanism for activation of NRs includes that the binding of ligands to the receptor induces the C-terminal AF2 helix to position in the active conformation [19]. The AF2 helix can then form a charge clamp pocket, completed by helices α3, α3', α4, and α5, which allows the receptor to interact efficiently with coactivator proteins [19,44–46]. In the ligand-free crystal structure of the COUP-TFII LBD, the AF2 helix does not form the charge clamp pocket but instead adopts an inactive conformation by occupying the coactivator binding site, thereby preventing the binding of coactivator proteins. This inactive conformation of COUP-TFII is facilitated by the kink of helix α10, which induces the last two turns of the C-terminal region of helix α10 to fit tightly into the ligand binding pocket. The collapse of helix α10 into the ligand binding pocket has also been observed in the inactive conformation of several other NRs. The CAR antagonist androstanol induces a similar kink of helix α10 from its straight agonist-bound conformation [47,48]. The apo-RXR structure also has its C-terminal portion helix α10 bent into the RXR ligand binding pocket [26,27]. It is interesting to note that the C-terminal portion helix α10 has been proposed as part of allosteric networks that transmit ligand binding signal across the dimer interface of NR [49,50]. Thus structural changes of the C-terminal part of helix α10 may represent a more general phenomenon involved in switching/modulating the activation function of NRs.

The autorepressed conformation of COUP-TFII AF2 helix has also been observed in two previous crystal structures of NR LBDs. The structure of the ligand-free tetramer of RXRα shows an autorepressed orientation where the AF2 helix protrudes away from the core domain and spans into the coactivator binding site in the adjacent monomer of the symmetric dimer [27]. Although this interaction is between two monomers, the RXRα AF2 helix physically excludes coactivator binding in a manner similar to that found in the structure of autorepressed COUP-TFII. The overall route mean square deviation (RMSD) for the 116 Cα atoms that align between the core of the LBD structures (α3, α4, α5, α7, α8, α9, and α10 to the Val373 kink, including loops) is 1.436 Å, which indicates a high degree of similarity between the autorepressed structures of COUP-TFII and RXRα and perhaps a conservation of transcriptional repression based on their structures. The main difference between the two structures, aside from the relative positioning of the AF2 helix, is the size of the ligand-binding pocket. As mentioned earlier, the COUP-TFII binding pocket in its ligand-free structure is virtually nonexistent and filled with two turns of the C-terminal half of α10 as well as hydrophobic and aromatic side chains. In contrast, the ligand-binding pocket of the RXRα tetramer is I-shaped and can crystallize with an alternative trans-isomer of retinoic acid [27]. Helix α3 of COUP-TFII is shorter than that of RXRα and folds closer to the center of the ligand-binding pocket, which creates a smaller pocket in COUP-TFII. In addition, the kink in COUP-TFII α10 occurs more N-terminally than does the separation of α10 and α11 in RXRα (V373 versus H435, respectively), which allows the C-terminal half of α10 to occupy deeper into the ligand-binding pocket of COUP-TFII than RXRα.

The antagonist-bound ERα structures also share similarity to the structure of COUP-TFII with the relative positioning of the AF2 helix [21,24]. The binding of OHT to ERα promotes a conformation of the AF2 helix that inhibits the binding of coactivators or corepressors. The ERα AF2 helix mimics the hydrophobic interactions of the coactivator peptide with a stretch of residues that resembles a coactivator peptide (LLEML instead of LXXLL, where the underlined residues are identical or similar to leucine (Figure 6). Identical to the structure of COUP-TFII, the N-terminal residue of the NR charge-clamp in ERα (K362) interacts with the C-terminal turn of the AF2 helix, making hydrogen bonds to the carbonyls of M543 and L544. This interaction between AF2 and the body of the NR LBD suggests that there may be conservation of interactions required to block the binding of either apo-NRs or antagonist-bound NRs with coactivators or corepressors.

### The Role of Dimerization in COUP-TFII Function

The COUP-TFII crystal structure is a dimer in which two monomers interact along the same interface, previously identified as important in homo- and heterodimerization of other NRs [24,26–28,46,51]. The majority of intermolecular interactions are mediated by residues from the N-terminal halves of helix α10, with two leucine residues forming the hydrophobic core of the interface. The L364A/L365A double mutant created to disrupt the dimer interface caused an 80% reduction in COUP-TFII function (Figure 3B) and reinforces the notion that COUP-TFs function as homodimers [34,35]. The dimeric structure and cell-based activation assays presented here thus provide additional insight into the roles of dimerization in COUP-TFII–mediated transcription activation. Interestingly, the residues involved in COUP-TFII dimerization are highly homologous to those found in the RXR dimer interface (Figure 6). It is possible that these residues are crucial in mediating COUP-TF heterodimer interactions with other NRs in addition to its homodimer.

COUP-TF has also been shown to serve as a repressor of transcription by directly binding to the LBD of NRs, a process termed transrepression [6,52,53]. This model of transrepression by COUP-TF involves the DNA-independent heterodimerization of COUP-TF LBDs with other receptors, such as TR, RAR, or RXR, and thus preventing these receptors from activating transcription. Although the specific details of this mechanism are unknown, one hypothesis is that once COUP-TF heterodimerizes with other LBD, they can either suppress the activation functions of these receptors or diminish their ligand-binding abilities by locking them in an inactive conformation [53]. The dimer structure of COUP-TFII solved in a ligand-free conformation fits this model of transrepression (Figure 1). In the absence of ligands, COUP-TFII is able to homodimerize along α7, α9, the N-terminal portion of helix α10, and the loop between α8 and α9 with its dimer interface resembling RXR homodimers and heterodimer interface [26, 27]. Conceivably, COUP-TFII would be able to heterodimerize with the unliganded forms of NRs, such as RXRα, through this same dimer interface and act as a transrepressor of RXRα function by blocking the ability of these receptors to interact with ligands and/or cofactors and subsequently inhibiting transcription. Thus the interaction between ligand-free, autorepressed conformation of COUP-TFII and other members of the NR2 subfamily may be a plausible explanation of how COUP-TFII can act as a repressor of transcription via the above model of transrepression.

### COUP-TFII as a Possible Retinoid Acid–Activated Receptor

Since COUP-TFI was first cloned nearly two decades ago, it has been puzzling whether the COUP-TF orphan NRs are ligand-regulated [54]. Despite the absence of a known ligand for COUP-TF, biological roles of this subfamily of NRs have been extensively studied. The structural and biochemical works presented in this paper have established that COUP-TFII is a ligand-regulated receptor, whose function can be activated by micromole concentrations of retinoic acids. This conclusion is supported by the following evidence. The first and the most important observation is the contrast between the autorepressed conformation in the apo-COUP-TFII structure and the ability of retinoic acids to promote COUP-TFII to interact with coactivators. The AF2 helix in the apo-structure of COUP-TFII occupies the coactivator binding site, thus physically blocking the receptor's ability to interact with coactivators. This is consistent with our AlphaScreen results (Figure 7B), which show that COUP-TFII is not able to interact with coactivator LXXLL motifs in the absence of ligand. In contrast, both 9cRA and ATRA are able to promote COUP-TFII to interact with the SRC-3 LXXLL motifs, suggesting that these ligands are able to reshape the AF2 conformation to accommodate the binding of coactivators. The second evidence is the ability of COUP-TFII to activate the *NGFI-A* reporter in multiple cell lines, which can be further potentiated by exogenous coactivators that require intact LXXLL coactivator motifs. The full activity of COUP-TFII is dependent on the intact structure of the COUP-TFII dimer, the charge clamp pocket for coactivator binding, and the residues that line the COUP-TFII ligand binding pocket (Figures 2–6). These data suggest that the mode of COUP-TFII activation is similar to the general model of NR activation, in which ligand binding induces the AF2 helix to form a charge clamp pocket to interact with LXXLL motifs of coactivators. The final evidence is that the “constitutive” activity of COUP-TFII in multiple cell lines is dependent on serum used in the assays. Charcoal-treated serum, which removes hydrophobic ligands such as steroids or retinoids in the serum, severely reduces COUP-TFII activation levels (Figure 7A). In contrast, the addition of retinoid acids elevates COUP-TFII activation (Figure 7D). Together, these data provide coherent evidences that support the conclusion that COUP-TFII is a ligand-regulated NR, where retinoid acids could serve as low-affinity ligands. Although retinoic acids may not be the physiologically relevant ligands for COUP-TF, because the concentrations of retinoic acids required for COUP-TFII activation is significantly higher than the endogenous levels of retinoic acids, our results nevertheless establish that the COUP-TF orphan receptors are ligand-regulated. Interestingly, COUP-TFII activates the *NGFI-A* reporter above the no-receptor control even with charcoal-stripped serum or in the absence of exogenous ligands (Figure 7A and 7D), indicating there are likely to be endogenous ligands produced in cultured cells. Identification of the endogenous ligands will be crucial for understanding the ligand-dependent pathways of COUP-TF. In addition, our data also provide a structural model of COUP-TF activation, in which ligand activation is mediated in part by releasing the receptor from its autorepressed conformation. Given that both vitamin A and the COUP-TF orphan receptors share many similar and important roles in development, the identification of COUP-TFII as a low-affinity retinoic acid receptor presented here provides a new window to look into the physiological relationship between these two previously unconnected pathways.

## Materials and Methods

### Protein preparation.

The human COUP-TFII LBD (residues 173–414 with C174S mutation located in the loop prior to helix α1 in the LBD) was expressed as a 6x Histidine-GST fusion protein from the expression vector pET24a (Novagen). BL21 (DE3) cells were grown to an OD_600_ of approximately 1.0 and induced with 50 μM of isopropyl-beta-D-thiogalactopyranoside (IPTG) at 16 °C. Six liters of cells were harvested and resuspended in 200-ml extract buffer (10 mM Tris pH 7.3, 200 mM NaCl, and 10% glycerol) and approximately 50 μg lysozyme, 0.1% triton X-100, 1 mM dithiothreitol (DTT), and 100 μM PMSF were added. Cells were passed through a French Press with the pressure set at 1,000 Pa, and lysate was centrifuged at 20,000 rpm for 30 min. The supernatant was added over a pre-equilibrated 25-ml glutathione-sepharose 4 fast flow column (Amersham Biosciences). The column was washed with 200 ml of wash buffer (10 mM Tris pH 8.0, 1 M NaCl, 10% glycerol, and 0.1% triton X-100) followed by buffer A (300 ml of 10 mM Tris pH 8.0, 100 mM NaCl, and 10% glycerol). The protein was eluted using buffer A supplemented with 4 mM reduced glutathione. The 6x Histidine-GST-COUP-TFII fusion protein was cleaved overnight with thrombin (0.5 NIH units/mg fusion protein) at 4 °C. The cleaved COUP-TFII protein was loaded onto a pre-equilibrated 10 ml Ni^2+^ chelating sepharose column (Amersham Biosciences) and eluted at ∼8% buffer B (500 mM imidazole in 10 mM Tris pH 8.0, 1 M NaCl, 10% glycerol). Ethylenediamine tetraacetic acid (EDTA) and DTT were added to 1 mM and protein was concentrated for crystallization. A typical yield of the purified COUP-TFII LBD was about 2 mg/l of cells.

### Crystallization and data collection.

Crystals of the COUP-TFII LBD were grown at 20 °C in hanging drops containing 3.0 μl of the above protein solution and 1 μl of well buffer containing 1.3 M or 1.5 M imidazole pH 5.6 and 1% Pluronic F68 detergent (Hampton). Small crystals (50 μm) appeared within 1 wk and grew to approximately 100–300 μm in size over the course of 3 wk. COUP-TFII crystals were crosslinked using glutaraldehyde and soaked in increasing concentrations of glycerol in the above well buffer. Iodine derivatives were soaked in the mother liquor solution supplemented with 250 mM NH_4_I, 25 mM Tris, and 35% glycerol. All crystals were flash frozen in liquid nitrogen before data collection.

The COUP-TFII crystals formed in the C2 space group with *a* = 97.85 Å, *b* = 47.76 Å, *c* = 43.13 Å, α = γ = 90°, and β = 100.87° (Table 1). The iodine datasets were collected with a MAR225 CCD detector at the at the ID line of sector-5 at the Advanced Photon Source at Argonne National Laboratory (Argonne, Illinois, United States). The observed reflections were reduced, merged, and scaled with DENZO and SCALEPACK in the HKL2000 package [55].

### Structure determination and refinement.

SHARP [56] was used to calculate initial phase information, and autoBUSTER [57] was used to auto-build an initial model of the COUP-TFII LBD. Quanta (Accelrys) was used to manually build the protein model followed by iterative refinement cycles with CNS [58] and REFMAC [58]. REFMAC was used for final refinement of the COUP-TFII structure, which include all residues except for 13 residues between α1 and α3, 17 residues between α5 and α6, and the C-terminal seven residues. The pocket volumes were calculated with the program voidoo using program default parameters and a probe with a radius of 1.2 Å [30] and surface areas were calculated with areaimol from the CCP4 suite of programs [59]. All figures were prepared using PyMOL [60].

### Transient transfection assays.

The expression plasmids of the mouse COUP-TFII, PGC1-α and SRC1–3, and the *NGFI-A* (−168/+33) promoter luciferase reporter in pXP2 were previously described [31]. All mutant COUP-TFII and SRC-3 plasmids were created by using the QuickChange Kit (Stratagene). For the GAL4-COUP-TFII chimera experiments, the COUP-TFII LBD construct (144–414) was cloned into the pBind vector and cotransfected with the pG5-Luc reporter (Promega). COS-7 and HEK-293T cells were maintained in DMEM containing 10% fetal bovine serum (FBS) and CHO-K1 cells were maintained in α-MEM containing 10% FBS. Cells were transiently transfected in DMEM or α-MEM supplemented with 5% FBS and 1 mM nonessential amino acids by using Lipofectamine 2000 (Invitrogen) according to the manufacturer's protocol. 24-well plates were inoculated with 75,000 cells 24 h prior to transfection. Each well of cells was transfected in Opti-MEM with 200 ng of reporter plasmid and 5 ng of *Renilla* luciferase expression plasmid phRL-CMV (Promega) in all experiments. COS-7 cells were used for all experiments except in Figure 2A. For coactivator experiments, cells were transfected with 100 ng COUP-TFII expression vector and 200 ng of either wild-type or mutant coactivators. For wild-type and mutant COUP-TFII transfections, 200 ng of DNA was used in each experiment. 24–30 h after transfection, cells were harvested and firefly and *Renilla* luciferase activities were measured.

For ligand activation assay, 50,000 COS-7 cells were plated in a 24-well plate 24 h before transfection. Cells were transiently transfected with 50 ng COUP-TFII expression vector, 150 ng of reporter plasmid and 0.5 ng of phRL-CMV (Promega). Medium was changed and compounds (all-trans retinoic acid and 9-cis retinoic acid) were added 14 h after transfection. Cells were incubated for another 24 h and harvested for luciferase assay by using Dual-Luciferase Reporter Assay System (Promega). Firefly luciferase values were normalized to *Renilla* luciferase, which was used as an internal transfection control. All assays were performed in triplicate. For statistical analysis, the fold induction was compared to wild type COUP-TFII (except when noted) using a Student's *t*-test (*p < 0.05, **p < 0.01, and ***p < 0.001).

### Ligand binding assays.

Ligand binding to COUP-TFII was determined by the ability of the ligands to promote COUP-TFII to recruit coactivator peptides, which was measured by an AlphaScreen kit (Perkin Elmer) as described for other NRs [30]. COUP-TFII LBD protein was purified as a 6X His-GST fusion protein for the assays. The experiments were conducted with approximately 0.4 μM receptor LBD and 0.1 μM of biotinylated SRC3–1 peptide (AENQRGPLESKGHKKLLQLLTSS) in the presence of 20 μg/ml donor and acceptor beads in a buffer containing 50 mM MOPS, 50 mM NaF, 50 mM CHAPS, and 0.1 mg/ml bovine serum albumin, all adjusted to a pH of 7.4. To screen for a potential ligand, 9-cis-retinoic acid (Sigma Aldrich), all-trans-retinoic acid (BioMol), dexamethasone (Sigma Aldrich), cortisol (Sigma Aldrich), and progesterone (Sigma Aldrich) were added to a concentration of 50 μM. EC50 values for 9cRA and ATRA were determined from a nonlinear least-square fit of the data based on an average of three repeated experiments, with standard errors typically less than 10% of the measurements.

## Abbreviations

9cRA: 9-cis retinoid acid
AF2: activation function-2
COUP-TF: chicken ovalbumin upstream promoter-transcription factors
DBD: DNA-binding domain
ERα: estrogen receptor α
LBD: ligand-binding domain
NR: nuclear receptor
